# CPR-IR is an insulin resistance index that is minimally affected by hepatic insulin clearance—A preliminary research

**DOI:** 10.1371/journal.pone.0197663

**Published:** 2018-05-23

**Authors:** Tsuyoshi Okura, Risa Nakamura, Yohei Fujioka, Sonoko Kawamoto-Kitao, Yuichi Ito, Kazuhisa Matsumoto, Kyoko Shoji, Keisuke Sumi, Kazuhiko Matsuzawa, Shoichiro Izawa, Etsuko Ueta, Masahiko Kato, Takeshi Imamura, Shin-ichi Taniguchi, Kazuhiro Yamamoto

**Affiliations:** 1 Division of Cardiovascular Medicine, Endocrinology and Metabolism, Department of Molecular Medicine and Therapeutics, Tottori University Faculty of Medicine, Yonago, Tottori, Japan; 2 School of Health Science, Tottori University Faculty of Medicine, Yonago, Tottori, Japan; 3 Division of Molecular Pharmacology, Tottori University Faculty of Medicine, Yonago, Tottori, Japan; 4 Department of Regional Medicine, Tottori University Faculty of Medicine, Yonago, Tottori, Japan; Shanghai Diabetes Institute, CHINA

## Abstract

**Background:**

Increased hepatic insulin clearance (HIC) is important in the pathophysiology of type 2 diabetes mellitus (T2DM). The aim of this study is to analyze an effective insulin resistance (IR) index that is minimally affected by HIC.

**Methods:**

Our study involved 20 participants with T2DM and 21 healthy participants without diabetes (Non-DM). Participants underwent a meal tolerance test from which plasma glucose, insulin and serum C-peptide immunoreactivity (CPR) were measured, and HOMA-IR and HIC were calculated. Participants then underwent a hyperinsulinemic-euglycemic clamp from which the glucose disposal rate (GDR) was measured.

**Results:**

The index CPR-IR = 20/(fasting CPR × fasting plasma glucose) was correlated more strongly with GDR, than was HOMA-IR, and CPR-IR could be used to estimate GDR. In T2DM participants with HIC below the median, HOMA-IR and CPR-IR were equally well correlated with GDR. In T2DM with high HIC, CPR-IR correlated with GDR while HOMA-IR did not. In Non-DM, CPR-IR and HOMA-IR were equally well correlated with GDR regardless of HIC. The mean HIC value in T2DM was significantly higher than that of Non-DM.

**Conclusions:**

CPR-IR could be a simple and effective index of insulin resistance for patients with type 2 diabetes that is minimally affected by HIC.

## Introduction

Insulin resistance and impaired insulin secretion are the core pathophysiologic defects in type 2 diabetes mellitus (T2DM) [1]. The glucose clamp test is the most accurate method of evaluating insulin resistance [2], however, this technique is very burdensome to the patients. Therefore, we generally use the homeostasis model assessment of insulin resistance (HOMA-IR) index obtained from glucose clamp test in daily clinical practice and in clinical research [3]. However, a past study reported that the adequacy of HOMA-IR may be limited in the patients with a low body mass index, decreased insulin secretion, and higher levels of fasting glucose, characteristics that are not uncommon in non-obese Korean patients with T2DM [4]. A past Japanese study reported that about half of Japanese patients with T2DM have some genetic background to the T2DM, and their insulin secretion is often impaired in lean patients [5]. Another study also reported that Japanese and Asian patients tend to have impaired insulin secretion [6], this background may limit the validity of the HOMA-IR in Asian populations.

The pancreas secretes equimolar ratio insulin and C-peptide in humans [7]. As C-peptide, unlike insulin, is not cleared by liver, insulin secretion can be accurately determined from the peripheral venous c-peptide concentrations [8]. Therefore, these results suggested that peripheral venous C-peptide concentrations more accurately reflect pancreatic insulin secretion ability than peripheral venous insulin concentrations. We previously reported that an insulin resistance index based on C-peptide levels, i.e., C-peptide immunoreactivity insulin resistance (CPR-IR) = 20/(fasting C-peptide immunoreactivity (CPR) × fasting plasma glucose) [9], may be more reliable than indexes based on insulin levels, including HOMA-IR and insulin sensitivity index (ISI) [10].

About half of the insulin that enters the liver is cleared in the average person [11]. The solute carrier family 30 member 8 gene (SLC30A8) is disease susceptibility gene of type 2 diabetes mellitus [12]. SLC30A8 encodes zinc transporter-8 (ZnT8), and it delivers zinc ions from the cytoplasm into insulin granules [13]. ZnT8 knockout (ZnT8KO) mice shows increased hepatic insulin clearance (HIC) which was assessed by the C-peptide to insulin ratio, and postprandial hyperglycemia [14]. These results suggest that increased HIC is important in the pathophysiology of type 2 diabetes mellitus.

Based on these results, we hypothesized that patients with type 2 diabetes mellitus with high HIC would show a weak correlation between HOMA-IR and glucose clamp outputs. Moreover, because C-peptide is not cleared by the liver, we predicted that the C-peptide-based index CPR-IR would be more accurate than insulin-based indexes. However, the muscle insulin sensitivity should be depend on the insulin concentration that may be reduced by the hepatic insulin clearance. According to these backgrounds, we thought that we need the glucose clamp study with evaluating the hepatic insulin clearance. In this study, we performed a meal tolerance test (MTT) and a glucose clamp in Japanese patients with type 2 diabetes mellitus and healthy volunteers without diabetes, and calculated HIC, glucose disposal rate (GDR) and the insulin resistance indexes CPR-IR, HOMA-IR, QUICKI, ISI and clamp-like index (CLIX).

## Methods

### Subjects

Fourteen males and six females with type 2 diabetes mellitus (T2DM participants) participated in this study at Tottori University Hospital between 2013 and 2015. We diagnosed T2DM by using the criteria of the World Health Organization [15]. We excluded the patients with liver disease, pancreatic disease, malignancy, or those taking corticosteroids from this study. We also excluded patients with serum creatinine > 1.3 mg/dl according to a past report, because these patients often show high serum C-peptide concentrations [16]. Ten T2DM participants were on diet therapy alone and 10 were using oral hypoglycemic agents including dipeptidyl peptidase inhibitors (10 participants), α-glucosidase inhibitors (two participants), sulfonylurea (two participants), and biguanides (two participants). None of the T2DM participants were using thiazolidinediones, glinides, sodium-dependent glucose transporter -2 inhibitors or insulin. Thirteen male and eight female healthy volunteers without diabetes (Non-DM participants) were also recruited for this study. None of the Non-DM participants had type 2 diabetes mellitus or were taking medications of diabetes. Participant characteristics from the T2DM and Non-DM groups are given in Table 1. All participants were examined using the protocols reported in our previous study [9].

**Table 1:** Participant characteristics.

	T2DM	Non-DM	P value
*n*	20	21	
Sex (male/female)	14/6	13/8	
Age (years)	56.7 ± 12.1	34.0 ± 9.1	<0.001
BMI (kg/m^2^)	27.1 ± 4.2	21.9 ± 2.9	<0.001
Waist circumstance (cm)	94.5 ± 13.6	78.0 ± 9.3	<0.001
Fasting plasma glucose (mmol/L)	7.08 ± 1.02	4.78 ± 0.45	<0.001
HbA1c (%)	7.15 ± 0.84	5.35 ± 0.27	<0.001
HbA1c (mmol/mol)	(54.6)	(35.0)	
eGFR (mL /min/1.73m^2^)	78.8 ± 23.0	94.7 ± 15.2	<0.05
Creatinine (mg/dl)	0.78 ± 0.22	0.72 ± 0.16	0.19
AST (IU/L)	31.3 ± 19.1	20.0 ± 6.5	<0.05
ALT (IU/L)	46.4 ± 37.0	22.0 ± 16.0	<0.05
Gamma-GTP (IU/L)	47.6 ± 44.3	23.3 ± 12.4	<0.05
LDL-C (mmol/L)	3.30 ± 0.78	3.04 ± 0.52	N.S.
HDL-C (mmol/L)	1.16 ± 0.30	1.75 ± 0.38	<0.01
TG (mmol/L)	1.60 ± 0.76	0.94 ± 0.54	<0.05
HOMA-IR	3.77 ± 2.34	1.73 ± 1.08	<0.005
CPR-IR	5.08 ± 2.74	11.3 ± 3.86	<0.001
QUICKI	0.32 ± 0.03	0.36 ± 0.02	<0.005
ISI	4.55 ± 3.21	6.99 ± 3.15	<0.05
CLIX	8.15 ± 4.13	12.4 ± 5.46	<0.001

Data are means ± standard deviation. T2DM, study participants with type 2 diabetes mellitus; Non-DM, study participants without diabetes; HbA1c, hemoglobin A1c; HOMA-IR, homeostasis model assessment of insulin resistance; QUICKI, Quantitative insulin sensitivity check index; ISI, insulin sensitivity index; CLIX, clamp-like index; CPR-IR, C-peptide immunoreactivity insulin resistance.

This study was conducted according to the principles expressed in the Declaration of Helsinki. The Ethics Committee of the Faculty of Medicine, Tottori University approved this study (approval number G161). We obtained informed consent from all of the participants using a procedure approved by the Ethics Committee.

### Meal tolerance test

The participants came to our hospital in the morning after fasting for at least 12 hours. The participants had a test meal devised by the Japan Diabetes Society (460 kcal/1882 kJ; 50% carbohydrate, 15% protein, 35% fat; and 1.6 g salt) [17]. The participants took orally their daily medications before and after the meal. We measured plasma glucose, serum insulin, and serum CPR at 0 (fasting), 30, 60, and 120 min after the test meal. We measured plasma glucose by using the glucose oxidase method. We also measured serum insulin and CPR levels by using chemiluminescent immunoassays (human insulin and CPR chemiluminescent immunoassay kits; Kyowa Medix, Tokyo, Japan). We measured HbA1c by high-performance liquid chromatography. We converted HbA1c percentage values to International Federation of Clinical Chemistry values (mmol/mol) using the HbA1c converter made by the National Institutes of Diabetes and Digestive and Kidney Diseases [18].

### Euglycemic-hyperinsulinemic clamp

We performed glucose clamps method 2 days after the MTT. The details of glucose clamp test were previously reported [9]. Briefly, the euglycemic-hyperinsulinemic clamp was performed by using an artificial endocrine pancreas (STG 55; Nikkiso, Shizuoka, Japan) to evaluate insulin sensitivity [2]. We performed a protocol of a primed constant infusion of insulin (100 mU/m^2^/min), and maintain plasma glucose levels at 5.2 mmol/L (95 mg/dL). According to the past study, this method achieved the steady-state plasma insulin level at 1200 pmol/L in patients with T2DM [19]. The steady-state glucose infusion rate (GIR) during 90–120 min was measured, the mean GIR during this time was defined as glucose disposal rate (GDR), which was used as a marker of peripheral insulin sensitivity.

We consider that a GDR > 10.0 mg/kg·min was normal at an insulin infusion rate of this protocol [20], and a GDR < 5.0 mg/kg·min was obviously insulin-resistant according to previous reports [21].

We also calculated the ratio M/I is a measure of the quantity of glucose metabolized per unit of plasma insulin concentration and is thus a reasonable index of tissue sensitivity to insulin [2]. We defined the M value as GDR, and I value as steady state insulin concentration. For convenience of data expression, we have multiplied the M/I ratio by 1200.

### Calculation of indices

HOMA-IR [3] = {[fasting plasma glucose (mmol/L)] × [fasting insulin (pmol/L)] } / 135.

QUICKI [22] = 1 / {log[fasting plasma glucose (mg/dL)] + log[fasting insulin (μU/mL)]}.

ISI [10] = 10,000 / square root {[fasting plasma glucose (mmol/L) × fasting insulin (pmol/L)] × [mean glucose × mean insulin during the MTT]}.

CLIX [23] = [serum creatinine (mg/mL) (× 0.85 if male) ] / {(mean area under the curve (AUC) glucose (mg/dl) × mean AUC C-peptide (ng/mL)} × 6600.

CPR-IR [9] = 20/[fasting-CPR (nmol/L) × fasting plasma glucose (mmol/L)] or 1080/[fasting-CPR (ng/mL) × fasting plasma glucose (mg/dl)]

HIC [24] = the ratio of the incremental areas under the MTT curve (AUC C-peptide 0–120 min/AUC insulin 0–120 min)

### Statistical analysis

Data are expressed as means ± standard deviation of the mean. We determined correlations between parametric clinical variables and GDR by using Pearson’s correlation analysis. We also calculated the intraclass correlation coefficient (ICC) to assess the agreement or consistency between pairs of indices. We calculated the partial correlation coefficients between GDR and CPR-IR with adjustment for the type of hypoglycemic agent/diet therapy. The partial correlation coefficients were defined as partial R. We also calculated the partial correlation coefficients between M/I value and CPR-IR and HOMA-IR with adjustment for age and BMI. We made the threshold of high or low HIC by the median value of HIC. We assessed differences in the mean value of HIC between T2DM and Non-DM participants by using an unpaired t-test. Values of *P* < 0.05 were considered significant. SPSS software version 24.0 (SPSS, Chicago, IL, USA) was used for all analyses.

## Results

For the T2DM participants, during the steady-state period of the glucose clamp, the mean GDR was 5.31 mg/kg·min and the mean insulin level was 1293 ± 326 pmol/L, the mean glucose level was 5.19 mmol/L (93.5mg/dl). For the Non-DM participants, during the steady-state period of the glucose clamp, the mean GDR was 9.64 mg/kg·min and the mean insulin level was 1109 ± 258 pmol/L, the mean glucose level was 5.07 mmol/L (91.4mg/dl). Mean plasma glucose, insulin and CPR levels from the MTT are given in Table 2. Mean values for HOMA-IR, QUICKI, ISI, CLIX, and CPR-IR calculated from the MTT are given in Table 1.

**Table 2:** Results of the meal tolerance test.

	Time (min)	0	30	60	120
	Glucose (mmol/L)	7.08 ± 1.02	9.30 ± 1.70	11.27 ± 2.40	10.30 ± 2.40
T2DM	Insulin (pmol/L)	71.2 ± 42.8	216.7 ± 165.4	324.9 ± 244.2	313.5 ± 180.9
	CPR (nmol/L)	0.71 ± 0.33	1.20 ± 0.61	1.62 ± 0.80	2.00 ± 0.79
	Glucose (mmol/L)	4.78 ± 0.45	6.59 ± 0.80	6.50 ± 1.81	5.48 ± 0.62
Non-DM	Insulin (pmol/L)	48.0 ± 26.5	280.2 ± 175.3	351.3 ± 175.3	187.5 ± 113.1
	CPR (nmol/L)	0.42 ± 0.16	1.16 ± 0.45	1.69 ± 0.53	1.30 ± 0.42

Data are means ± standard deviation. T2DM, study participants with type 2 diabetes mellitus; Non-DM, study participants without diabetes; CPR, C-peptide immunoreactivity

**Fig 1:**
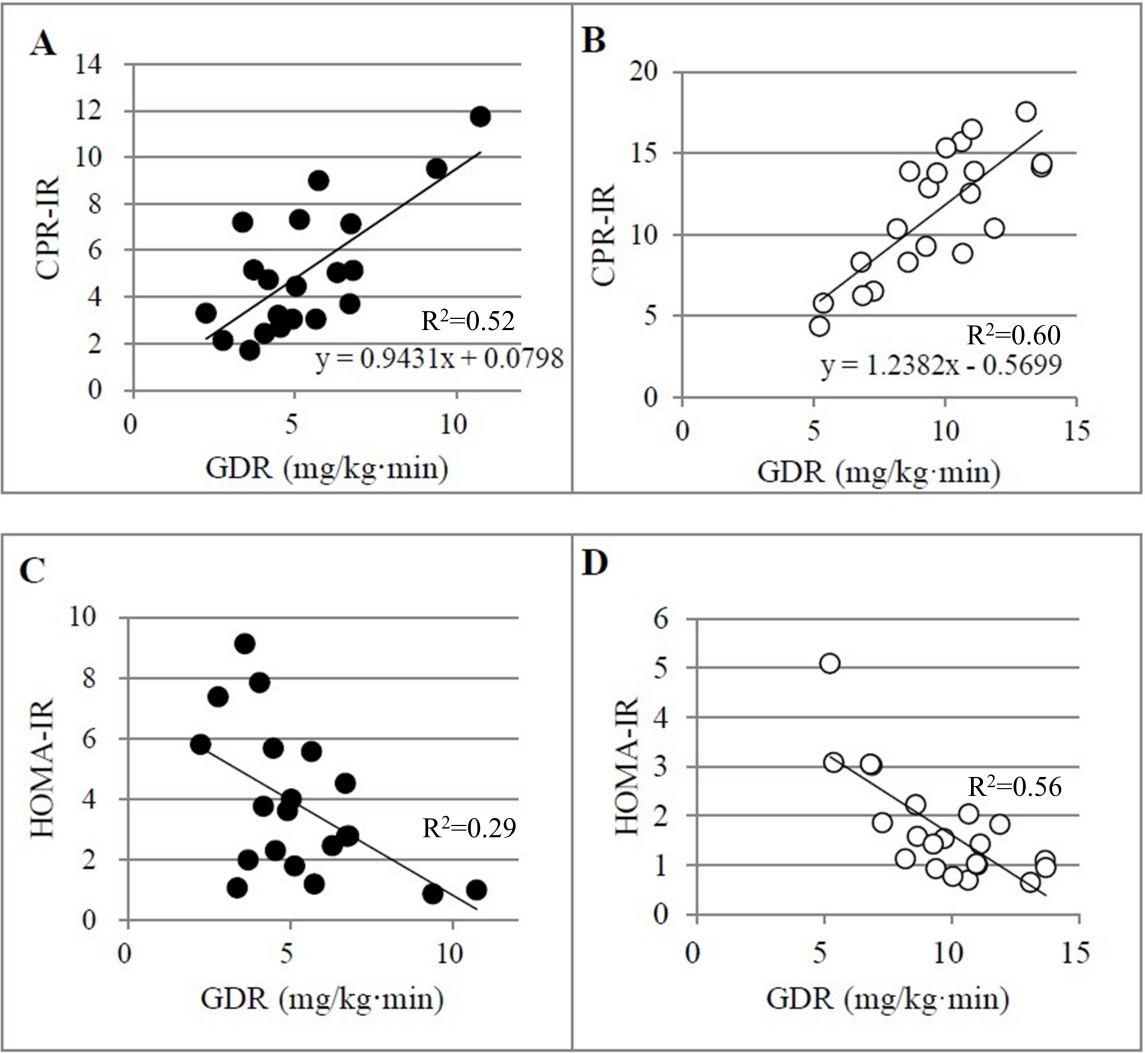
The correlation between the glucose disposal rate (GDR) and C-peptide immunoreactivity insulin resistance (CPR-IR; A, B) or homeostasis model assessment of insulin resistance (HOMA-IR; C, D), where black circles depict results from study participants with type 2 diabetes mellitus and white circles depict results from study participants without diabetes.

Across all T2DM participants, the CPR-IR index was strongly correlated with GDR (Table 3), and CPR-IR could be used to estimate GDR using the regression equation given in Fig 1A. HOMA-IR (Table 3, Fig 1C), QUICKI, ISI and CLIX were also correlated with GDR (Table 3).

**Table 3:** Correlation coefficients between indices of insulin resistance and glucose disposal rate.

Index	All T2DM		T2DM HIC<6.0		T2DM HIC>6.0	
	(*n* = 20)		(n = 10)		(n = 10)	
	*r*	*P*	*r*	*P*	*r*	*P*
HOMA-IR	−0.54	< 0.005	-0.68	< 0.05	−0.21	0.55
CPR-IR	0.72	< 0.0005	0.69	< 0.05	0.64	<0.05
QUICKI	0.58	< 0.001	0.74	< 0.05	0.37	0.29
ISI	0.61	< 0.005	0.78	< 0.01	0.50	0.14
CLIX	0.62	< 0.005	0.82	< 0.005	0.42	0.21
**Index**	**All Non-DM**		**Non-DM HIC<5.0**		**Non-DM HIC>5.0**	
	**(*n* = 21)**		**(n = 11)**		**(n = 10)**	
	*r*	*P*	*r*	*P*	*r*	*P*
HOMA-IR	−0.75	< 0.0005	-0.76	< 0.01	−0.70	<0.05
CPR-IR	0.78	< 0.0005	0.82	< 0.005	0.73	<0.05
QUICKI	0.73	< 0.0005	0.72	< 0.05	0.70	<0.05
ISI	0.68	< 0.005	0.76	< 0.01	0.65	<0.05
CLIX	0.56	< 0.01	0.48	0.13	0.61	0.05

Correlation coefficients were determined using Pearson’s product moment correlation coefficient test. T2DM, study participants with type 2 diabetes mellitus; Non-DM, study participants without diabetes; HOMA-IR, homeostasis model assessment of insulin resistance; QUICKI, Quantitative insulin sensitivity check index; ISI, insulin sensitivity index; CLIX, clamp-like index; CPR-IR, C-peptide immunoreactivity insulin resistance.

We also analyzed the ICC between specific indices and GDR. The ICC between GDR and CPR-IR was 0.66. ICC between GDR and HOMA-IR was -0.58, for QUICKI was -0.73, for ISI was 0.52, for CLIX was 0.28, respectively. We determined partial correlation coefficients between CPR-IR and GDR after adjusting for the use of hypoglycemic drugs/diet therapy. The correlation between CPR-IR and GDR remained significant in all of the analyses, partial R for biganide was 0.679 (P<0.005), for sulfonylurea was 0.677 (P<0.005), for α-glucosidase inhibitors was 0.681 (P<0.005), for dipeptidyl peptidase inhibitors was 0.677 (P<0.005), and for diet therapy alone was 0.677 (P<0.005), respectively.

Across all Non-DM participants, the CPR-IR index was strongly correlated with GDR (Table 3), and CPR-IR could be used to estimate GDR using the regression equation given in Fig 1B. HOMA-IR (Table 3, Fig 1D), ISI and CLIX were also correlated with GDR (Table 3). The ICC between GDR and CPR-IR was 0.61. ICC between GDR and HOMA-IR was -0.91, for QUICKI was -0.87, for ISI was 0.37, for CLIX was 0.26, respectively.

The median value of HIC in T2DM participants was 6.0. In the 10 T2DM patients with low HIC (< 6.0), CPR-IR, HOMA-IR, ISI and CLIX were all correlated with GDR (Table 3). However, in the 10 T2DM participants with high HIC (> 6.0), CPR-IR was correlated with GDR, while HOMA-IR, ISI and CLIX were not (Table 3).

The median value of HIC in Non-DM participants was 5.0. Non-DM participant CPR-IR and HOMA-IR were equally well correlated with GDR in the participants with high HIC (> 5.0) (Table 3).

The mean value of HIC was significantly higher in T2DM participants than Non-DM participants (T2DM 7.0 ± 1.2, Non-DM 5.5 ± 6.3; un-paired t- test *P* < 0.05).

In the T2DM participants with high HIC, mean BMI was significantly lower than that of the participants with low HIC (high HIC 25.2 ± 5.7, low HIC 29.8 ± 2.3; un-paired t-test P<0.05). The mean age of the T2DM participants with high HIC was significantly higher than that of the participants with low HIC (high HIC 64.0 ± 11.1, low HIC 49.4 ± 6.8; un-paired t-test P<0.005). Moreover, the high HIC participants showed higher glucose levels than the low HIC participants, however, there were no significance (fasting plasma glucose 7.18 ± 1.09 vs. 7.05 ± 0.88 mmol/L; P = 0.38, 2hr postprandial glucose 11.04 ± 2.61 vs. 9.49 ± 2.09 mmol/L; P = 0.08, HbA1c 7.37 ± 0.62 vs. 6.97 ± 0.870%; P = 0.14).

We also analyzed M/I value and adjusted by age and BMI. There was strong correlation between M/I and CPR-IR in the T2DM patients with high HIC (Table 4).

**Table 4:** Partial correlation coefficients between indices of CPR-IR, HOMA-IR and M/I value adjusted by age, BMI.

**Index**	**All T2DM**		**T2DM HIC<6.0**		**T2DM HIC>6.0**	
	**(*n* = 20)**		**(n = 10)**		**(n = 10)**	
	*r*	*P*	*r*	*P*	*r*	*P*
HOMA-IR	−0.64	< 0.005	-0.77	< 0.05	−0.65	0.07
CPR-IR	0.75	< 0.001	0.68	0.06	0.76	<0.05
**Index**	**All Non-DM**		**Non-DM HIC<5.0**		**Non-DM HIC>5.0**	
	**(*n* = 21)**		**(n = 11)**		**(n = 10)**	
	*r*	*P*	*r*	*P*	*r*	*P*
HOMA-IR	−0.38	0.11	-0.35	0.34	−0.47	0.24
CPR-IR	0.45	< 0.05	0.42	0.26	0.57	0.13

The partial correlation coefficients were determined with adjustment for age and BMI. T2DM, study participants with type 2 diabetes mellitus; Non-DM, study participants without diabetes; HOMA-IR, homeostasis model assessment of insulin resistance; CPR-IR, C-peptide immunoreactivity insulin resistance.

Of the 10 Non-DM participants with normal range GDR > 10.0, the mean GDR was 11.7 and the mean CPR-IR was 13.9.

## Discussion

This study shows that the C-peptide-based index CPR-IR [20/(fasting CPR × fasting plasma glucose)] was more strongly correlated with GDR than were the insulin-based indexes HOMA-IR, QUICKI, ISI, and the C-peptide-based CLIX in T2DM participants with high HIC. In our study, HOMA-IR was not correlated with GDR in T2DM participants with high HIC. C-peptide, unlike insulin, is not cleared by the liver, resulting in a longer half-life: 34 minutes and 4 minutes for CPR and insulin, respectively [7, 8]. Accordingly, we predicted that the C-peptide-based index would perform better than the insulin-based indexes. These results suggest that plasma insulin levels were affected by HIC and plasma C-peptide levels were a better indicator of insulin bioactivity in skeletal muscle, as measured by the glucose clamp technique.

Our study showed that HOMA-IR, QUIKI, and ISI were significantly correlated with GDR in Non-DM participants with high HIC, but not with GDR in T2DM participants with high HIC. This may be because the mean HIC of T2DM participants was significantly higher than that of Non-DM participants. In addition, it has been shown in mice that ZnT8 expression is downregulated in the early stages of diabetes [25], suggesting that many patients with hyperglycemia may suffer from dysregulated insulin clearance. These results are suggesting that hyperglycemia associates HIC, suggesting that the CPR-IR index may be more reliable than insulin-based insulin resistance indexes in people with type 2 diabetes mellitus. We consider that HOMA-IR is suitable for Non-DM participants and mild diabetes, because HIC is not high, however, it might not be suitable for hyperglycemic participants.

In this study, CPR-IR was also more closely correlated with GDR than was CLIX, which is also based on C-peptide. In particular, CLIX was not correlated with GDR in T2DM participants with high HIC. This may be because CLIX uses postprandial glucose and C-peptide levels, which are more affected by insulin secretion ability than insulin resistance. Moreover, it has been shown that human carriers of a major risk allele for SLC30A8, exhibit increased HIC and higher postprandial glucose levels than their respective controls [14]. The high HIC affects postprandial glucose and insulin levels, and may thus affect peripheral insulin sensitivity and CLIX. Based on this logic, we predict CPR-IR to be more reliable than CLIX as a measure of insulin resistance. A further benefit of CPR-IR is that its measurement requires only a single blood sample and no MTT or oral glucose tolerance tests (OGTTs).

HOMA-IR is the most widely used as insulin resistance index, but may be of limited use in patients with a lower body mass index, decreased beta cell function, and high fasting plasma glucose levels [4]. East Asian populations such as the Japanese and Koreans often show decreased beta cell function, so HOMA-IR may be a less reliable estimate of insulin resistance in these subjects [5, 6]. Chiu et al. reported that Caucasians were more insulin sensitive than Asian-Americans and that beta cells made up for differences in insulin sensitivity [26]. Moreover, Hsu et al. reported type 2 diabetes in Asian-Americans have insulin resistance despite their low BMI [27]. We predict that our CPR-IR index could be more suited to East Asian populations than HOMA-IR. Moreover, our study showed that the mean BMI in the T2DM participants with high HIC was significantly lower than that of the participants with low HIC. The decrease of HIC showed a trend for association with a risk of incident metabolic syndrome [28]. These results suggested that HIC is the important pathophysiology of glucose metabolism and obesity.

Our study had some limitations. The comparatively small number of participants (total 41, T2DM 20) and the fact that the age and BMI of the Non-DM participants did not match those of the T2DM participants suggests that our study require confirmation with larger subjects. We calculated sample size using sample size calculator [29], effect size α = 0.05, power (1-β) = 0.8, r = 0.50, each group need 28 participants about high and low HIC groups. Therefore, we consider that our study eventually needs 56 patients with type 2 DM and 56 non-DM participants, total 112 participants. However, the glucose clamp method is a burdensome test, and it is hard to enter the T2DM patients with high glucose levels with no medications, and older non-DM participants with obese, it will take a long period for the confirmation of the study. Therefore, we decided to add “a preliminary research” in the title. Moreover, in the T2DM participants with high HIC, mean BMI was significantly lower than that of the participants with low HIC. The mean age of the T2DM participants with high HIC was significantly higher than that of the participants with low HIC. We therefore analyzed M/I value and adjusted by age and BMI. There was strong correlation between M/I and CPR-IR in the T2DM patients with high HIC. However, CPR-IR and HOMA-IR showed not so high correlation with M/I value in non-DM subjects. These results suggested that age and BMI may affect the HIC and M/I value, the age and BMI matched study is needed. Conversely, CPR-IR might be an index that is minimally affected by age and BMI.

The T2DM participants were taking diabetes medications at the time of the study that varied in their nature, and it is possible that these different medications affected their insulin and C-peptide responses in the MTT, especially in the patients with a sulphonylurea. Glucagon-like peptide-1 reduces the clearance of endogenously-released insulin, and may thus affect insulin levels by increasing prehepatic insulin secretion and reducing insulin clearance [30]. Because half of the T2DM participants in our study were taking dipeptidyl peptidase 4 inhibitors, these participants may show low HIC. However, 7 T2DM participants with DPP4 inhibitor showed HIC > 6.0 (data not shown), therefore DPP4 inhibitors might not affect HIC so much. However, many oral hypoglycemic agents could change insulin clearance. For example, metformin increases insulin clearance [31]. We also excluded the patients with renal dysfunction, renal dysfunction affects the C-peptide levels because C-peptide is also degraded by the kidney and cleared by the kidney. In this study, eGFR levels were different between DM and non-DM group, however, serum creatinine levels were not different in each group. The eGFR level is greatly affected by age, therefore, we consider renal function were the same level in each group. However, if we use our index in patients with renal dysfunction, a corrective may be needed in the equation, as with CLIX.

Using our insulin infusion protocol, the steady-state plasma insulin level was previously reported to be 1200 pmol/L in patients with type 2 diabetes mellitus [19]. This insulin level completely suppresses the hepatic glycogenolysis, though many studies have used an insulin infusion protocol of 600 pmol/L. This study also did not analyze the risk allele of SLC30A8, rs1366642. We are currently conducting a larger study with age and BMI matched Non-DM participants and T2DM participants that do not take diabetes medications that use an insulin infusion protocol of 600 pmol/L and involves an analysis of the genome of SLC30A8. Because ISI and CLIX were developed from OGTTs, we must note that differences between consuming a test meal and a pure glucose load may also affect glucose and insulin levels. MTT were used in our study as OGTTs are best avoided in patients with severe diabetes because of the risk of hyperglycemia. Because CLIX and ISI calculated with the data obtained with MTT, both clinical utility and phathophysiological significance of these indexes are totally unknown. However, some studies used the HIC obtained from MTT [32], we consider our study was acceptable. The steady state plasma insulin during hyperinsulinemic euglycemic clamp in healthy subjects is lower than that in type 2 diabetes patients in the present study. It may be suggesting that insulin clearance is decreased in type 2 diabetes patients. Consistently, many previous studies reported that type 2 diabetes and insulin resistant subjects have decreased insulin clearance. However, an index of insulin clearance using MTT data showed completely opposite result. The standard deviation value of steady state plasma insulin during hyperinsulinemic euglycemic clamp was large, we suspect that insulin clearance was decreased type 2 DM, however insulin clearance was increased in the patients with high post prandial glucose levels as previous report [14]. However, it is possible that the HIC index might be no longer precise method to compare the insulin clearance level in Japanese type 2 diabetes. Although we would like to investigate hepatic insulin clearance, insulin clearance by using glucose clamp data is also important. Unfortunately, we did not measure the C-peptide levels at steady state of glucose clamp in this study. We would like to investigate insulin clearance by using glucose clamp data in the future study. Importantly, CPR-IR accurately reflected the level of insulin resistance determined using a glucose clamp, despite variability in the hypoglycemic medications taken by our T2DM participants. Although there are these limitations, we consider that our results and the CPR-IR index may contribute daily clinical practice and large clinical study of patients with T2DM, as CPR-IR is easy to obtain from measurements taken from a fasting blood sample.

The results of this study suggest that CPR-IR = 20/(fasting CPR × fasting plasma glucose) is a simple and useful index of insulin resistance, and may be more reliable than HOMA-IR for Japanese patients with type 2 diabetes mellitus. Particularly, CPR-IR showed a stronger correlation with GDR than did HOMA-IR in T2DM participants with high hepatic insulin clearance. Furthermore, there is a possibility to estimate GDR from CPR-IR using a regression equation. CPR-IR could be an index of insulin resistance that is minimally affected by hepatic insulin clearance.
